# Introduction of the 2005 cardiopulmonary resuscitation guidelines did not increase return of spontaneous circulation in a physician-staffed prehospital emergency medical system

**DOI:** 10.1186/cc9717

**Published:** 2011-03-11

**Authors:** G Gemes, S Wallner, G Wildner, M Rigaud, G Prause

**Affiliations:** 1 Medical University of Graz, Austria; 2 Austrian Red Cross, Graz, Austria

## Introduction

Cardiopulmonary resuscitation (CPR) guidelines published by the European Resuscitation Council are intended to improve survival of cardiac arrest by implementing medical practice based on scientific findings. This study investigated whether the introduction of the 2005 CPR guidelines, which mandated several fundamental practice changes, improved the rate of return of spontaneous circulation (ROSC) in a physician-staffed prehospital emergency medical system.

## Methods

Emergency physician protocol sheets from calls responding to cardiac arrest were reviewed and the following data were collected: bystander CPR and bystander use of a semi-automatic defibrillator, medication administered by emergency physicians, number of defibrillations, on-the-scene thrombolysis, occurrence of ROSC. These parameters were compared in a 3-year period from each before and after the introduction of the 2005 CPR guidelines.

## Results

A total of 632 CPR protocols were analyzed, and the groups were comparable regarding age, sex, delay and initial rhythm. Bystander CPR was observed in 35% of the cases, with no difference between before and after the introduction of the 2005 guidelines and was not associated with an increase in ROSC. Bystander use of a defibrillator was rare (2.5%), but was associated with an increase in ROSC. When advanced life support by emergency physicians was conducted according to the 2000 guidelines, ROSC occurred in 29% of the cases, whereas ROSC occurred in 36% of the cases after 2005 (*P *= 0.058). Adrenaline and manual defibrillations were applied less frequently after 2005, whereas amiodarone and atropine were used more frequently. The application of thrombolysis was not different before and after 2005, but was associated with an increase in ROSC.

## Conclusions

In our setting, the 2005 CPR guidelines apparently failed to reach out to laypersons, as bystander CPR was neither more frequent nor associated with an increase in ROSC. The 2005 guidelines had an impact on advanced life support practice by emergency physicians, but there was only a trend to an increase in ROSC.

